# Association of 'Klotho' gene polymorphism with cerebral infarction

**DOI:** 10.5937/jomb0-34196

**Published:** 2022-04-08

**Authors:** Yu Li, Qiang Zhang, Haiping Bao, Chen Nie

**Affiliations:** 1 The Second Affiliated Hospital of Dalian Medical University, Department of Neurology, Dalian, China; 2 Dalian Shipyard Rehabilitation Hospital, Department of Rehabilitation Medicine, Dalian, China

**Keywords:** Klotho, cerebral infarction, polymorphism, Klotho, cerebralni infarkt, polimorfizam

## Abstract

**Background:**

We aimed to investigate the expression of Klotho gene in peripheral blood of patients with cerebral infarction (CI) and the association of its polymorphisms with the occurrence of CI.

**Methods:**

A total of 60 CI patients (CI group) and 20 healthy people receiving physical examination (control group) were enrolled as the research subjects. The expression of Klotho gene in CI group and control group was determined using enzyme-linked immunosorbent assay kit. Single nucleotide polymorphisms (rs192031, rs200131 and rs102312) in the promoter region of the Klotho gene were typed via conformational difference gel electrophoresis. Besides, whether the distribution frequencies of Klotho genotypes conformed to Hardy-Weinberg equilibrium was evaluated by chi-square test. Meanwhile, the associations of Klotho alleles and gene polymorphisms with CI occurrence were analyzed.

**Results:**

The protein expression level of Klotho in the peripheral blood was remarkably lower in patients in CI group than that in control group (P<0.05).HardyWeinberg equilibrium analysis revealed that Klotho gene polymorphisms (rs192031, rs200131 and rs102312) conformed to the genetic equilibrium distribution (P>0.05). Gene-based association analysis manifested that only rs192031 polymorphism and alleles were correlated with CI occurrence (P<0.05). Systolic blood pressure and highdensity lipoprotein cholesterol were notably higher in CI patients with TT genotype of Klotho gene polymorphism rs192031 than those in control group (P<0.05). Furthermore, there were no associations of rs200131 and rs102312 polymorphisms and alleles with the occurrence of CI (P>0.05).

**Conclusions:**

The expression level of Klotho is evidently reduced in the peripheral blood of CI patients. Rs192031 in the promoter region of the Klotho gene is associated with the occurrence of CI, while rs200131 and rs102312 have no relations with CI.

## Abstract (sr)

**Uvod:**

Cilj nam je bio da istražimo ekspresiju Klotho gena u perifernoj krvi pacijenata sa cerebralnim infarktom (CI) i povezanost njegovih polimorfizama sa pojavom CI.

**Metode:**

Ukupno 60 pacijenata sa CI (CI grupa) i 20 zdravih osoba na fizikalnom pregledu (kontrolna grupa) upisano je kao subjekti istraživanja. Ekspresija Klotho gena u CI grupi i kontrolnoj grupi je određena korišćenjem kompleta za en - zimski imunosorbentni test. Polimorfizmi pojedinačnih nukleotida (rs192031, rs200131 i rs102312) u promotorskom regionu Klotho gena su tipizovani putem konformacione razlike u gel elektroforezi. Osim toga, da li su frekvencije distribucije Klotho genotipova u skladu sa Hardy-Wein-bergovom ravnotećom procenjeno je hi-kvadrat testom. U međuvremenu, analizirane su asocijacije Klotho alela i polimorfizama gena sa pojavom CI.

**Rezultati:**

Nivo ekspresije proteina Klotho-a u perifernoj krvi bio je značajno niži kod pacijenata u CI grupi nego u kontrolnoj grupi (P<0,05). Hardy-Weinbergova analiza ravnoteže otkrila je da se polimorfizmi Klotho gena (rs192031, rs200131 i rs102312) podudaraju sa distribucija genetičke ravnoteže (P>0,05). Analiza asocijacije zasnovana na genima pokazala je da su samo rs192031 polimorfizam i aleli u korelaciji sa pojavom CI (P<0,05). Sistolni krvni pritisak i holesterol lipoproteina visoke gustine bili su značajno viši kod pacijenata sa CI sa TT genotipom polimorfizma Klotho gena rs192031 od onih u kontrolnoj grupi (P<0,05). Štaviše, nije bilo asocijacija rs200131 i rs102312 polimorfizama i alela sa pojavom CI (P>0,05).

**Zaključak:**

Nivo ekspresije Klotho-a je evidentno smanjen u perifernoj krvi pacijenata sa CI. Rs192031 u promotorskom regionu Klotho gena je povezan sa pojavom CI, dok rs200131 i rs102312 nemaju veze sa CI.

## Introduction

Cerebral infarction (CI) is an ischemic-hypoxic necrosis induced by insufficient blood supply to local brain tissues. It is characterized by high incidence and disability rates. Various factors can lead to the occurrence of CI, the main cause of which is cerebral atherosclerosis [1]. The nature of atherosclerosis lies in the chronic activation of endothelial cells caused by inflammatory and fibro proliferative reactions, which can induce vascular stenosis and insufficient blood supply to the brain, and the secondary rupture of fibrous cap in atherosclerotic plaque eventually leads to CI [2] [3].The correlation of genetic factors (especially gene polymorphisms) with acute atherosclerotic CI has become a research hotspot in recent years. Cathepsin S (CTSS), a cysteine protease of the papain superfamily, plays a vital role in the degradation and reconstruction of extracellular matrix, antigen presentation, inflammation, immunity and angiogenesis [4]. A study revealed that single nucleotide polymorphisms (SNPs) (rs774320676 and rs928508030) of the CTSS gene are related to the risk of acute atherosclerotic CI. The T allele of rs774320676 and the G allele of rs928508030 of CTSS are genetic susceptibility genes for acute atherosclerotic CI [5].

*Klotho*, as an anti-aging gene, is able to reduce oxidative stress, thus protecting the cardio-cerebrovascular system [6]. A study indicated that the elevated plasma Klotho concentration in patients with acute ischemic stroke is correlated with a good functional prognosis [7]. Basic experiment illuminated that *Klotho* is an inducer of metabolic coupling between neurons and astrocytes. *Klotho* can be released by the neuronal glutamatergic activity and insulin regulation, thereby stimulating the formation and release of lactic acid in astrocytes [8]. Traditional Chinese medicine ligustilide is capable of ameliorating cerebral ischemia-reperfusion injury in mice by up-regulating *Klotho* expression [9]. However, the association of *Klotho* gene polymorphism with CI has not been reported yet.

The distribution of polymorphisms (rs192031, rs200131 and rs102312) in the promoter region of the Klotho gene was determined in CI patients in this study, so as to provide a certain reference for further research of the genetic pathogenesis of CI.

## Materials and Methods

### Subjects

A total of 60 CI patients admitted to our hospital from January 2016 to January 2019 were enrolled as the research subjects, including 31 males and 29 females aged (57.41±2.34) years old. About 4 μL of venous blood was extracted, anticoagulated with sodium citrate, and stored in a refrigerator at -20°C. Mean while, 20 healthy people receiving physical examination were selected as the controls, with 10 males and 10 females aged (57.13±2.19) years old. This study was approved by the Ethics Committee of Linyi Central Hospital, and all the participants signed the informed consent. The patients in the CI group met the diagnostic criteria of the *Chinese Guidelines for Diagnosis and Treatment of Acute Ischemic Stroke 2019*, without transient ischemic attack, and the diagnosis was confirmed by imaging examinations. The subjects in the control group were healthy people who underwent routine physical examination in our hospital, without a history of cardiovascular and cerebrovascular diseases and related family history.

### Detection of serum Klotho protein

About 4 μL of venous blood was collected and anticoagulated with sodium citrate to detect serum Klotho protein using an enzyme-linked immunosorbent assay (ELISA) kit (Sigma-Aldrich, St. Louis, MO, USA) in accordance with the instructions.

### Deoxyribonucleic acid (DNA) extraction

A total of 4 μL of patient's EDTA-anticoagulated blood was taken to extract genomic DNA according to the instructions of the DNA Extraction Kit (Guge Bio-Technology Co., Ltd., Wuhan, China). Subsequently, the quality of 2 μL of sample was measured in 1.5% agarose gel electrophoresis. Meanwhile, the concentration of the extracted DNA was determined using an ultraviolet spectrophotometer.

### Polymerase chain reaction (PCR) amplification

Primers were designed to amplify *Klotho* gene polymorphismsrs192031, rs200131 and rs102312. Primer sequences of each polymorphism were shown in Table 1. The reaction system of PCR (20 μL) included 2.0 μL of DNA template, 10.0 μL of 2× mix, 0.4mL of forward primer, 0.4 μL of reverse primer, and 7.2 μL of ddH_2_O. PCR amplification was performed under the following conditions: 95°C for 120 s, 35 cycles of 94°C for 30 s, 57°C for 90 s and 72°C for 60 s, followed by extension at 72°C for 10 min. Subsequently, agarose gel electrophoresis was utilizedto detect the amplification of gene fragments.

**Table 1:** Primer sequences and product sizes of different polymorphisms in the promoter region of the *Klotho* gene

Polymorphism	Primer sequence (5’-3’)	Product (bp)
rs192031	Forward: AGCTGATGGCTATCGTAGCGACCReverse: TGGGCTAGCTAGCTAGTCGG	223
rs200131	Forward: AAGTCGATCGTTAGGGCAAReverse: GTGACTTAGGCCAATGAAA	302
rs102312	Forward: AGGCAAATTCGATCGTAGCTAGReverse: TGCTGTAGCTAGCTGATCG	381

### Ligase detection reaction

The upstream and downstream probes used in this reaction were designed and synthesized by BGI. The upstream probe was modified by phosphorylation at the 5'-terminal region to prepare a probe mixture with a concentration of 12.5 pmol/μL. Ligase detection reaction system (3.05 μL) was composed of 0.05 μL of ligase, 1 μL of buffer, 1 μL of PCR product, and 1 μL of probe mixture. PCR amplification was carried out under the following conditions: 95°C for 120 s, 94°C for 15 s and 50°C for 25 s, 30 cycles in total. After that, the concentration was measured using the ultraviolet spectrophotometer. Subsequently, BGI was commissioned to sequence and analyze the target gene. All data were analyzed using Gene Mapper (Table 2).

**Table 2:** Probe sequences and product sizes of ligase reaction for different polymorphisms of the *Klotho* gene

Polymorphism	Probe	Probe sequence (5’-3’)	Product (bp)
rs192031	rs192031 rs192031- Ars192031-T	P-ACGTAGCTAGCTAGTTTTTTTTTTTTTTTTTTT-FAM TTTTTTTTTTTTTTTACCCATTTTTTTTTAT TTTTTTTTTTTTTTTGCGACGAGCATTTTTTTTTAAA	124
rs200131	rs192031 rs192031-C rs192031-T	P-AGCCATGCACCCAATTTTTTTTTTTTTTTTTTT-FAM TTTTTTTTTTTTTTTTTTTTTTTTTCGTAGCTAAAC TTTTTTTTTTTTTTTTTTTTTTTTTTACGATCGATG	115
rs102312	rs102312 rs102312-Ar s102312-C	P-ACGGGATGCCATTTTTTTTTTTTTTTTTTT-FAM TTTTTTTTTTTTTTTTTTTTTTTTTTGCGGACGAG TTTTTTTTTTTTTTTTTTTTTTTTGCGGCCAAA	108

### Statistical analysis

Statistical Product and Service Solutions (SPSS) 22.0 software (IBM, Armonk, NY, USA) was applied for statistical analysis. Enumeration data were expressed by frequency and percentage, and measurement data were presented as mean±standard deviation. The genotype frequency in the sample was calculated and tested using the Hardy-Weinberg equilibrium formula. The chi-square test was used for multiple comparisons of enumeration data. Besides, *t*-test and analysis of variance were utilized for measurement data. P<0.05 indicated that the difference was statistically significant.

## Results

### Comparisons of clinical baseline data between CI group and control group

As shown in Table 3, blood glucose and systolic blood pressure were obviously increased, but highdensity lipoprotein cholesterol (HDL-C) significantly declined in CI group compared with those in control group (P<0.05).

**Table 3:** Comparisons of clinical data between CI group and control group

Index	Control group	CI group	P
Triglyceride (mmol/L)	1.75±0.28	1.81±0.21	0.112
Total cholesterol (mmol/L)	4.88±0.48	4.91±0.25	0.341
HDL-C (mmol/L)	1.28±0.11	1.14±0.49	0.003
LDL-C (mmol/L)	2.99±0.28	3.02±0.12	0.288
Blood glucose (mmol/L)	5.22±1.28	6.29±0.94	0.001
Systolic blood pressure (mmHg)	131.92±4.02	145.03±6.82	0.000
Diastolic blood pressure (mmHg)	80.02±3.02	84.29±4.02	0.018

### Comparison of Klotho protein levels in peripheral blood between CI group and control group

**Figure 1:**
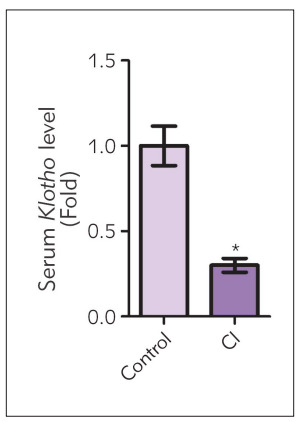
Comparison of *Klotho* protein levels in peripheralblood between CI group and control group CI: cerebral infarction group. Control: healthy control group. *P<0.05: a statistical difference vs. control group

As shown in Figure 1, the expression level of Klotho in the peripheral blood of patients was markedly reduced in CI group in comparison with that in control group (P<0.05), indicating that Klotho might be involved in the occurrence and development of CI.

### Analysis results of Klotho gene polymorphism-srs192031, rs200131 and rs102312

The *Klotho* gene polymorphisms rs192031, rs200131 and rs102312 in CI group and control group were cleaved by *BSTU I* restriction enzyme, manifesting that polymorphism rs192031 had two alleles (A and T) and three genotypes (AA, AT and TT), rs200131 had two alleles (C and T) and three genotypes (CC, CT and TT), and rs102312 had two alleles (A and C) and three genotypes (AA, AC and CC).

### Results of Hardy-Weinberg equilibrium test

The linkage disequilibrium test results of different Klotho gene polymorphisms were tested using the Hardy-Weinberg equilibrium formula. As shown in Table 4, r^2^ <0.33 was detected between groups of polymorphisms, indicating the conformity of polymorphisms with the equilibrium test between groups.

**Table 4:** Results of linkage equilibrium test for the *Klotho* gene polymorphisms between groups

Polymorphism	r ^2^		
	rs192031	rs200131	rs102312
rs192031	-	0.012	0.108
rs200131	0.012	-	0.221
rs102312	0.108	0.221	-

### Associations of Klotho gene polymorphisms with CI

The genotype frequencies of each gene polymorphism in the two groups were shown in Table 5. The polymorphism rs192031 was remarkably related to the occurrence of CI (P<0.05), while rs200131 and rs102312 were not correlated with CI (P>0.05).

**Table 5:** Distribution of different genotypes of *Klotho* gene polymorphisms and CI

Group	rs192031			rs200131			rs102312		
	AA	AT	TT	CC	CT	TT	AA	AC	CC
CI	10.1%	50.9%	39.0%	20.1%	48.0%	30.0%	20.1%	50.9%	29.0%
Control	24.0%	51.2%	24.8%	22.8%	46.0%	31.2%	19.3%	51.2%	29.5%
C^2^	1.661			0.499			0.717		
P	0.032			0.221			0.610		

### Association of Klotho alleles with CI

According to the genotype frequencies of each polymorphism in the two groups (Table 6), the polymorphism rs192031 was obviously associated with occurrence of CI (P<0.05), while rs200131 and rs102312 had no relations with CI (P>0.05).

**Table 6:** Distribution of alleles of *Klotho* gene polymorphisms and CI

Group	rs192031		rs200131		rs102312	
	A	T	C	T	A	C
CI	30.00%	70.00%	70.21%	29.79%	45.23%	54.77%
Control	82.11%	17.89%	72.22%	27.78%	42.08%	57.92%
C^2^	1.432		0.782		0.644	
P	0.001		0.114		0.412	

### Correlations of TT genotype of Klotho gene polymorphism rs192031 with clinical parameters of CI

The further research revealed that the systolic blood pressure and HDL-C were notably higher in CI patients with TT genotype of *Klotho* gene polymorphism rs192031 than those in control group (P<0.05) (Table 7).

**Table 7:** Correlation analysis of TT genotype of *Klotho* gene polymorphism rs192031 and clinical parameters of CI

Index	TT genotype		P
	CI	Control	
Age	48±7	49±10	0.231
Triglyceride, (mmol/L)	1.88±0.27	1.85±0.18	0.302
Total cholesterol, (mmol/L)	4.91±0.26	4.91±0.18	0.429
HDL-C, (mmol/L)	1.33±0.28	1.16±0.15	0.019
LDL-C, (mmol/L)	3.02±0.15	2.98±0.21	0.192
Blood glucose, (mmol/L)	6.22±0.56	5.81±0.47	0.821
Systolic blood pressure, (mmHg)	142.11±4.20	134.83±5.22	0.001
Diastolic blood pressure, (mmHg)	82.11±5.92	80.01±6.92	0.231

## Discussion

CI is a cerebrovascular disease caused by cerebral blood supply disorders, which is characterized by high morbidity and high mortality, seriously threatening people's lives [10]. CI can be caused by multiple factors, especially atherosclerosis. Atherosclerotic CI is a multi-source disease resulting from the combined action of genetic and environmental factors. Atherosclerosis can lead to stenosis, occlusion and thrombosis of the blood vessel cavity, or the shedding of unstable plaques can result in CI [11]. Therefore, further elucidating the genetic mechanism of CI occurrence is of important significance for the early prevention and precise treatment of CI.

Previous studies have shown that the polymorphisms of multiple genes are potentially correlated with the occurrence and prognosis of CI. The allele frequency of APOE 4 is notably higher in CI patients than that in healthy controls [12]. Tissue inhibitors of metalloproteinases (TIMPs), as endogenous inhibitors of matrix metalloproteinases, participate in the normal cellular processes and the occurrence and progression of atherosclerosis. A study indicated that there is a strong linkage disequilibrium between 1296T/C and -915A/G of TIMP gene, and people with TC+ CC genotype are 1.8 times more likely to suffer mixed carotid plaque than those with TT genotype [13]. The certain association of *Klotho* gene polymorphisms with the occurrence and progression of CI was revealed in this study.

In animal models, a broad phenotype similar to the aging phenotype will be caused by suppressing the *Klotho* gene, including atherosclerosis, ectopic calcification, infertility, skin atrophy and severe hypoglycemia, while the overexpression of *Klotho* gene increases the overall life span of guinea pigs by 20-30% [14]. The human *Klotho* gene is located on chromosome 13 and can be expressed as a secretory *Klotho* protein by variable cleavage of the third exon. The anchored *Klotho* protein is mainly present in the distal convoluted tubules of the kidney and the choroid plexus of the brain, but it can be processed and released into the blood after translation, dissociating outside the cells and playing a hormone-like role [15]. *Klotho* gene has 6 SNPs in exon 2 and flanking regions, and such polymorphisms are closely related to the occurrence and progression of cardiocerebrovascular diseases [16]. The content of serum *Klotho* is higher in patients with a history of myocardial infarction but no history of coronary artery disease or stroke, but the haplotype of *Klotho* is not correlated with the above variables [17]. *Klotho* gene polymorphism may be a genetic risk factor for atherosclerotic coronary artery disease rather than vasospasm angina pectoris in the Japanese population. Specifically, a higher ratio of A genotype of the *Klotho* gene polymorphism G-395A was observed in patients with coronary heart disease than that in healthy controls [18].The correlation between polymorphisms (rs192031, rs200131 and rs102312) in the promoter region of *Klotho* and CI occurrence in the Han population was analyzed in this study. The protein was extracted from the peripheral blood of healthy people undergoing physical examination and CI patients. First, the results of ELISA revealed that the expression of Klotho protein was lower in the peripheral blood of patients in CI group (P<0.05). Subsequently, the target polymorphisms were genotyped to record the distribution of genotype and allele frequencies in different groups. The results indicated that there were significant correlations between the *Klotho* gene polymorphism rs192031 and its genotypes and the occurrence of CI (P<0.05). People with AT genotype were more likely to suffer CI than those with AA or TT genotype. The polymorphisms rs200131 and rs102312 and their genotypes had no remarkable associations with CI occurrence (P>0.05).

## Conclusions

In conclusion, this study illuminates for the first time that the *Klotho* gene polymorphism rs192031 was potentially associated with the occurrence of CI, while polymorphisms rs200131 and rs102312 and their genotypes were not related to the onset of CI.

## Dodatak

### Acknowledgements

No

### Conflict of interest statement

The authors reported no conflict of interestregarding the publication of this article. 
